# Multi-Class Detection of Neurodegenerative Diseases from EEG Signals Using Lightweight LSTM Neural Networks

**DOI:** 10.3390/s24206721

**Published:** 2024-10-19

**Authors:** Laura Falaschetti, Giorgio Biagetti, Michele Alessandrini, Claudio Turchetti, Simona Luzzi, Paolo Crippa

**Affiliations:** 1 Department of Information Engineering, Università Politecnica delle Marche, Via Brecce Bianche 12, I-60131 Ancona, Italy; g.biagetti@univpm.it (G.B.); m.alessandrini@univpm.it (M.A.); c.turchetti@univpm.it (C.T.); p.crippa@univpm.it (P.C.); 2 Neurology Clinic, Department of Experimental and Clinical Medicine, Università Politecnica delle Marche, Torrette, I-60126 Ancona, Italy; s.luzzi@staff.univpm.it

**Keywords:** Alzheimer’s disease (AD), classification, deep learning, electroencephalography, EEG, feature extraction, long short-term memory (LSTM), multi-class classification, neurodegenerative diseases, recurrent neural network (RNN)

## Abstract

Neurodegenerative diseases severely impact the life of millions of patients worldwide, and their occurrence is more and more increasing proportionally to longer life expectancy. Electroencephalography has become an important diagnostic tool for these diseases, due to its relatively simple procedure, but it requires analyzing a large number of data, often carrying a small fraction of informative content. For this reason, machine learning tools have gained a considerable relevance as an aid to classify potential signs of a specific disease, especially in its early stages, when treatments can be more effective. In this work, long short-term memory-based neural networks with different numbers of units were properly designed and trained after accurate data pre-processing, in order to perform a multi-class detection. To this end, a custom dataset of EEG recordings from subjects affected by five neurodegenerative diseases (Alzheimer’s disease, frontotemporal dementia, dementia with Lewy bodies, progressive supranuclear palsy, and vascular dementia) was acquired. Experimental results show that an accuracy up to 98% was achieved with data belonging to different classes of disease, up to six including the control group, while not requiring particularly heavy computational resources.

## 1. Introduction

The “World Population Prospects”, published by the United Nations, estimated that the total world population aged 65 and over reached 761 million in 2020 and is expected to double to almost 1.6 billion by 2050 (2022 Revision). Globally, the number of people aged 80 and over is growing even faster than the number of people aged 65 and over and will exceed the number of newborns (1 year of age or less) by the mid-2030s, reaching 265 million by then (2024 Revision) [1]. As a result, the impact on families and societies caused by the number of aging people is becoming increasingly significant. In particular, according to the World Health Organization, neurodegenerative age-related diseases, such as Alzheimer’s disease (AD), which are often characterized by a progressive decline in functional and cognitive abilities are increasing rapidly, becoming an alarming problem for the health sector.

Alzheimer’s disease (AD) and other neurodegenerative conditions such as frontotemporal dementia (FTD), dementia with Lewy bodies (LBD), and progressive supranuclear palsy (PSP), alongside non-degenerative conditions like vascular dementia (VAD), severely impact various aspects of a patient’s life, including cognitive functions, memory, and basic daily activities. These diseases affect millions of people, and their prevalence is expected to rise as life expectancy increases [2,3]. Diagnosing these diseases in their early stages, such as mild cognitive impairment, is crucial for effective treatment [4].

The electroencephalogram (EEG) is a non-invasive test that records brain electrical activity and has a wide field of applications in the medical area, one of which is the detection of neurodegenerative diseases. Recently, the EEG has gained prominence as a diagnostic tool due to its relative simplicity and non-invasiveness compared with more complex procedures like histological sampling or magnetic resonance imaging [5,6,7,8,9]. The EEG provides valuable, even if indirect, insights into a subject’s brain activity. However, analyzing EEG data can be challenging due to the potential overlap of symptoms between different pathologies or normal age-related changes, as well as the large number of data that require careful examination for specific patterns. Consequently, research has increasingly focused on applying machine learning algorithms to detect patterns in EEG signals, aiming to assist clinicians in accurately classifying a subject’s condition within a predefined set of diseases. A wide range of machine learning algorithms are used in the published literature on EEG classification studies for dementia detection [10,11,12]. Well-established methods such as support vector machines (SVMs), *k*-nearest neighbors (kNNs), logistic regression, and random Forests remain relevant in the classification of AD and other types of dementia [13,14,15]. However, deep learning methodologies have gained increasing popularity in classifying EEG signals in AD and further dementia research [16,17,18,19,20,21].

Most of the works in the literature deal with binary classification to distinguish between healthy subjects and those affected by Alzheimer’s, or to distinguish between healthy subjects and the preliminary stage of dementia, called mild cognitive impairment (MCI). Alvi et al. [22] proposed an LSTM framework for MCI detection from EEG signals, and designed 20 different LSTM models and investigated them with a publicly available MCI database, named the EEG MCI database [23], to find out the best one. The best model achieved 96.41% accuracy. A similar approach was previously proposed in Siuly et al. [24] to provide a robust biomarker for efficient detection of MCI patients using an extreme learning machine (ELM) neural network, achieving 98.78% accuracy on the same dataset. Previous attempts on the same dataset, such as the work by Yin et al. [25], achieved a 96.94% accuracy using a 3D evolution method for feature selection and a SVM as the classifier. However, this study involved a limited number of subjects (11 MCI and 11 healthy) and was computationally expensive due to its extensive pre-processing steps dedicated to the EEG signals denoising and features extraction. A machine learning method was also applied in Farina et al. [26] through a logistic regression algorithm on a custom dataset realized by collecting data related to resting state EEGs, structural MRIs (sMRIs), and rich neuropsychological data from older adults (55+ years) with AD or amnestic MCI (aMCI), as well as healthy controls (about 60 per group).

Although the acquired dataset contained multiple classes, in this case a binary classification was also performed by comparing the three pairs of classes: AD vs. N, MCI vs. N, and AD vs. MCI. In Ieracitano et al. [27], a dataset composed of 189 subjects (63 AD patients, 63 MCI patients, and 63 normal subjects) was acquired and a multi-layer perceptron (MLP) network was chosen as the classifier. Also in this case, the classification was principally applied to couple of classes (AD vs. N, AD vs. MCI; MCI vs. N), reducing to a binary classification. Only the case AD vs. MCI vs. N was investigated for the multi-class classification task. The proposed model achieved the highest accuracy of 96.95 ± 0.5% in AD vs. N, 90.24 ± 0.7% in AD vs. MCI, 96.24 ± 0.5% in MCI vs. N, and 89.22 ± 0.67% in AD vs. MCI vs. N classifications.

In a few other recent works, the two stages of the disease were examined (AD, MCI), in relation to healthy subjects (N), thus realizing a multi-class classification task with three classes. S. J. Ruiz-Gómez et al. [28] addressed a three-class classification problem by analyzing the performances of three methods, linear discriminant analysis (LDA), quadratic discriminant analysis (QDA) and an MLP artificial neural network, on a dataset composed of 111 subjects (37 AD patients, 37 MCI patients, and 37 N subjects) in order to discriminate AD, MCI, and N classes and develop a multi-class classifier. The overall accuracy of the models in the three-class classification task was 58.82% with LDA, 60.78% with QDA, and 62.75% with MLP, achieving better performance in pairwise class comparisons. Sharma et al. [29] analyzed eight EEG biomarkers (power spectral density, skewness, kurtosis, spectral skewness, spectral kurtosis, spectral crest factor, spectral entropy, fractal dimension) from 44 subjects in four conditions: eye-open, eye-close, finger tapping test, and continuous performance test. They achieved an accuracy for each event in the range from 73.4% to 89.8% for three binary classes.

In the mentioned related works, attention is paid to the accuracy in the precision of the classification without considering the number of parameters required by the network and the consequent memory occupation. These two aspects are fundamental if the network has to be implemented in a real-time, low-cost embedded system, but above all if we want to decrease the inference time. This aspect regards the research of networks with a reduced number of parameters, namely lightweight networks, still maintaining a good accuracy. As already mentioned, Alvi et al. in [22] investigated 20 different LSTM networks for MCI detection using the EEG MCI database, focusing on this aspect and reporting the number of nodes for each implemented LSTM network. The authors found the best model and tested for binary classification, in the configuration (1024, 512), that corresponded to a number of parameters exceeding one million, according to the equation $N_{parameters}=4*\left(\left(n_{input}+n_{output}\right)*n_{output}+n_{output}\right)$, where $n_{input}$ is the number of input units and $n_{output}$ is the number of output units (hidden units). This gives an estimation of what is considered a lightweight LSTM network in the literature. For comparison, our proposed network only requires about 5000 parameters. Searching for an LSTM lightweight network with a reduced number of parameters and memory occupation is one of the aims of the proposed method, together with maintaining a good accuracy both in binary and multi-class classification.

In our previous work [17], binary classification was investigated to distinguish AD subjects from healthy ones by using recurrent neural networks (RNNs), successfully resulting in over 97% accuracy on the test data (a subset of the dataset used in this work related to the two considered classes). Due to the nature of the EEG data, an RNN was considered for its ability to handle temporal dependencies. The main advantage of RNNs is their ability to model sequences of data, unlike traditional neural networks. This capability allows each pattern in the sequence to be influenced by previous patterns, making RNNs particularly effective for time-series data [30,31,32,33]. Specifically, an RNN with LSTM units was chosen for its ability to handle both long-term and short-term dependencies, addressing many computational and stability issues that can occur in RNNs, particularly with long sequences [34,35].

In the subsequent work [16], the design and the performance analysis of this type of network was extended to the multi-class case, taking into consideration four classes related to neurodegenerative pathologies, specifically AD, FTD, and LBD, together with normal subjects (N). In particular, an RNN was developed for the classification of neurodegenerative diseases from EEG recordings belonging to a custom dataset. The RNN processes the spectral representation of the data and utilizes LSTM layers at its core, following an artifact removal step to enhance network performance, achieving a global accuracy of 75.3%.

In this paper, an enhanced version of the previous method to perform multi-class classification of various neurodegenerative diseases with similar EEG recording characteristics using lightweight LSTMs is proposed. Several LSTMs were implemented and tested on different numbers of classes, grouped according to their relevance from a medical point of view:

- N, AD, FTD, LBD: to discriminate among the main degenerative dementias AD, FTD, and LBD [36,37,38], collectively called DDs;
- N, AD, FTD, LBD, PSP: to discriminate PSP [39], in addition to the DDs;
- N, AD, FTD, LBD, VAD: to try and discriminate VAD [40], in addition to the DDs;
- N, DD, VAD: the three DD classes are fused together.
- N, AD, FTD, LBD, PSP, VAD: this last test, that considered all the classes contained in the dataset, was conducted to validate the flexibility of the implemented networks, since it has no relevance from a medical point of view.

The second-last group compares the main degenerative dementias included in the single-class DD to VAD, which has different clinical causes. Degenerative dementias are caused by an unknown degenerative process, while vascular dementia is caused by a cerebral arteriosclerosis, which can determine multiple cortical and/or subcortical infarcts, strategic single infarcts, non-infarction white matter lesions, hemorrhages, and hypoperfusion as possible causes of VAD. Traditionally in neurology, vascular dementia is considered a “secondary dementia” (consequent to circulation problem), while the other forms of dementia are usually named “primary dementias”, i.e., dementia caused by a still unknown degenerative process.

As previously mentioned in a brief overview of the state of the art, these diseases have been analyzed with several machine learning methods but always as a binary classification, and, to the best of our knowledge, only in a few cases by adding a third class. Moreover, these works focus only on the accuracy performance without taking into account the computational complexity of the developed models, that is, important parameters to evaluate the deployability of the models in different devices, also with constrained resources and low-cost, low-power consumption characteristics. The proposed method outperforms the state-of-the-art results in terms of accuracy with a given number of classes, but above all it addresses the classification problem with a number of classes greater than three, still obtaining results superior or in line with the state of the art, maintaining a low computational complexity.

The rest of the paper is organized as follows. Section 2 describes the proposed methods, including the acquired dataset, the data pre-processing, and the design of the LSTM neural networks. Experimental results are presented in Section 3 and discussed in Section 4. Finally, some conclusions are drawn in Section 5.

## 2. Methodology

This work aims to obtain a compact and lightweight neural network for the classification of the signals, so after the canonical filtering, standardization, and windowing of the input sequences, these are converted into their spectral representation. The resulting set of features with which the neural network is trained no longer depends on time, but is expressed as a linear combination of a suitable ordered basis. It has already been demonstrated in our previous works, in [15] through a preliminary study using machine learning methods and in [17] using a first approach with deep-learning methods, that this change of representation often brings better results, while allowing a simplified network to still reach high accuracy.

To transform the input signals, the discrete Karhunen–Loève transform (DKLT) is used. This is a well-known technique [17,41] that is able to separate the temporal evolution of a signal from its statistical variations, resulting in an optimal representation. It is akin to a frequency-domain transform, where instead of pure sinusoids the basis is selected to best represent the given set of signals with the minimum number of free parameters.

Because of this, the number of features obtained through the DKLT can be truncated, using only its most significant values. This is called principal component analysis (PCA), and it simultaneously attains the reduction of the complexity of the data and the disposal of the less informative components that can worsen the classification, as are often associated with measurement noise. Such denoising effect can effectively replace other common pre-processing steps with the same purpose, like band-pass filtering.

**Figure 1:**
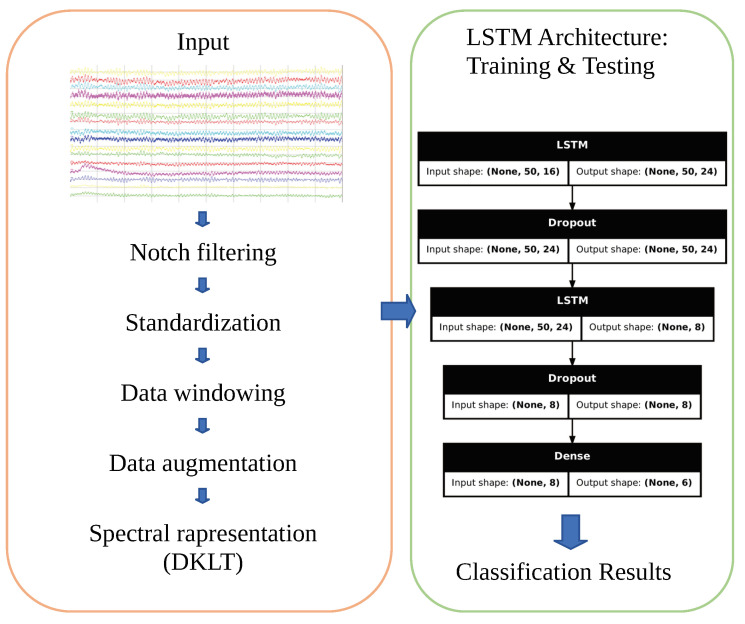
Full pipeline of proposed method.

In the following subsections, each step, starting with details on the data acquisition, will be discussed. Moreover, Figure 1 shows the full pipeline of the proposed method.

### 2.1. EEG Data Acquisition

A dataset comprising EEG recordings of five different neurodegenerative diseases (AD, FTD, LBD, PSP, VAD) was created by the Neurological Clinic, Department of Experimental and Clinical Medicine, at the Università Politecnica delle Marche. The dataset was obtained from subjects as part of a routine medical diagnosis in a hospital setting; the data were collected according to the Declaration of Helsinki, informed consent had been obtained at the time of original data collection, and all recorded data were adequately anonymized prior to being employed in this study.

Table 1 shows a summary of the consistency of the dataset, expressed in terms of total duration of the recordings for the six classes considered, specifically five different neurodegenerative diseases (AD, FTD, LBD, PSP, VAD), and the healthy condition (referred to as class N) used as the control group. In total, over 35 h of recordings were used in this study. The demographic distribution of the subjects (age, sex, education, and illness duration) was selected to be as similar as possible across the different groups.

The data were collected using a Galileo BE Plus PRO Portable, Light version, which is capable of 37 total connections, consisting of 22 unipolar and 8 bipolar AC/DC inputs. The electrodes were applied in the standard 10–20 configuration (the numbers being distances between adjacent electrodes, expressed as a percentage of the total available space on the subject’s skull, across the two directions). The EEG recordings were all sampled at 128 Hz, using between 21 and 23 tracks according to the clinical needs of the specific case. All signals from a given subject were acquired synchronously, but recordings from different subjects had naturally occurring different durations.

**Figure 2:**
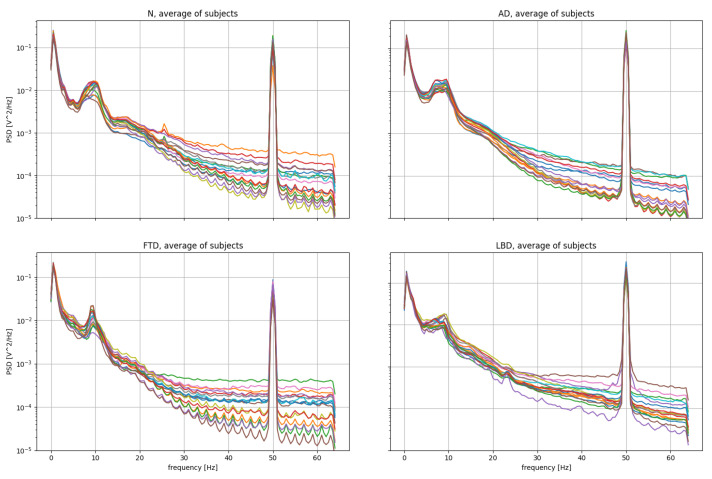
PSD of some of the EEG signals in the dataset (N, AD, FTD, LBD classes).

It is interesting to analyze the power spectral density (PSD) of the EEG signals (Figure 2). The PSD is computed with the Welch method [42], splitting the signal into overlapping segments and computing the squared magnitude of the discrete Fourier transform for each part. The final value of the PSD is computed from the average of the obtained values. It can be seen that there is a strong noise component at 50 Hz (electrical grid). As described in the following sections, we remove such components through a notch filter. Moreover, as already explained, the DKLT has the effect of removing the remaining noise components, leaving only the most significant components of the signal.

Apart from the obvious interference caused by the electrical grid, EEG signals are prone to several noise sources, especially in clinical settings. To estimate the specific level of noise in the dataset, we used the RELAX framework v2.0.0 [43,44], an extension to the well-known EEGLAB toolkit (v2024.2) for Matlab R2024b, specially oriented to EEG signals. By running an automated cleaning with the RELAX tool on a sample of the subjects’ data, the results show a signal-to-noise ratio ranging from 5 to 20 dB approximately. As stated, and because the cleaning methods are dependent on the specific setup used for acquisition, we chose to use the DKLT to isolate the principal components of the signal and hopefully remove, among others, the main noise sources.

### 2.2. Data Pre-Processing

#### 2.2.1. Data Preparation

Some pre-processing of the EEG data was required, due to the heterogeneity of the different track sets among different subjects and unavoidable variations in the signal acquisition conditions.

First, as not all subjects had the same track set being recorded, a subset of signals, common to all the subjects, was isolated. It consists of 16 tracks: Fp1, Fp2, F7, F3, F4, F8, T3, C3, C4, T4, T5, P3, P4, T6, O1, O2.

Then, the data were shaped in purely numerical matrices of size n×16, with *n* being the number of time points and 16 the number of selected (common) EEG tracks. Such data can be seen as time-based series of 16-dimensional points associated to each test.

#### 2.2.2. Filtering

A notch filter at 50 Hz was preliminarily applied to all the signals to remove a significant component produced by noise from the power line.

#### 2.2.3. Standardization

Since different signals, both in the same subject and among different patients, have significant variations in their magnitude, due to sensitivity to physical variations of the testing setup, statistical standardization was used to normalize each signal. Standardization consists in scaling a signal to obtain a final mean and standard deviation of 0 and 1, respectively, through the formula:

(1) $$y\left[n\right]=\frac{x[n]-\mu }{\sigma }$$

where μ and σ are the mean and standard deviation, respectively, of the original signal.

#### 2.2.4. Data Windowing

Input data from different subjects consist of a limited number of records with varying length. In order to obtain a substantial number of fixed-size segments to use as inputs to the PCA, and thus providing a statistically significant set of data, input signals are split into windows of fixed size *w* along the time axis. Windows also overlap by *o* samples, so that the *n*-th data window contains samples in the range $\left[(w-o)\;n\;,\;(w-o)\;n+w-1\right]$ of the original data.

Data windowing is also commonly performed in neural networks and other machine learning frameworks [17,31,45], with *w* and *o* being essential hyper-parameters to be established. Section 3 describes the values that were used.

Data windows from different tests are finally merged, so that the final dataset has the size N×w×16, where *N* is the total number of data windows.

#### 2.2.5. Data Augmentation

When the number of data inputs used to train the neural network are not equally distributed among all the different classes, a bias towards a specific category may result. The standard solution to this issue is data augmentation, i.e., generating more data for classes with the least amount of occurrences from what is already available.

In this work, the oversampling algorithm was used, where data from classes with a lower number of inputs are duplicated to increase the size of those classes. Moreover, to avoid perfectly identical sequences, some noise with a Gaussian distribution was added to the new data. Section 3 reports the chosen magnitude of the added noise.

In addition to a more balanced statistical distribution, the benefit of this kind of data oversampling is a larger number of data, provided that the dataset is limited in size. Adding some random noise to the duplicated data allows for a bigger variety in the input samples, useful for proper training of the neural network.

### 2.3. Spectral Representation

As mentioned, an essential step of this algorithm is converting the original data into a spectral representation using the DKLT. To this end, with X being a generic data matrix, where each row represents a sample, a standard method consists in using the singular value decomposition (SVD), such that

(2) $$X=USV^{T}$$

where S is a diagonal matrix of singular values, and U, V contain the singular vectors. Using that, a new matrix can be defined, namely

(3) K=XV

which expresses X as a set of features, in terms of the base represented by V, since X can be recovered as $X=KV^{T}$.

Then the principal component analysis (PCA) is performed. Only the most significant singular values are retained. (2) is usually constructed so that singular vectors are in decreasing order of magnitude so that the *p* most significant ones are associated to the first *p* columns of V. In this way, K can have fewer columns than X, with only the most representative components of the signals being retained, leading to lesser complexity.

In the case of the present work, a complication arises from the input data having more than two dimensions: the size is N×w×16 due to windowing (Section 2.2.4). In order to apply (2) and (3), the data are reduced to a bidimensional matrix, X, of size (16N)×w, and then the compressed matrix, K (which is (16N)×p), is reverted back to its original three-dimensional shape as follows:

(4) $$\begin{array}{c} R^{N\times w\times 16}\to R^{N\times 16\times w}\to R^{(16N)\times w}∋X\\ X\overset{\mathrm{DKLT}}{\to }K\\ K\in R^{(16N)\times p}\to R^{N\times 16\times p}\to R^{N\times p\times 16} \end{array}$$

where *p* is the number of components retained after the DKLT (p<w). The choice of the intermediate steps responds to the need of obtaining a conveniently sized matrix for the SVD, such that the resulting vector base is a statistically significant representation of a portion of any EEG trace.

### 2.4. Design of LSTM

The RNN used in this work consists of the layers listed in Table 2. The specific sizes shown refer to a truncation to p=50 components of the signal features, resulting in each input (data window) having a size of 50×16.

The core of the network is represented by two cascaded LSTM layers, having different numbers of neurons present in the two layers but with same “tanh” activation function for all the input layers. Each LSTM layer is followed by a dropout layer with 20% rate, randomly discarding part of the input in order to reduce overfitting. Overfitting is a common problem when training a DNN, occurring when the network adapts too closely to the training data, resulting in poor predictions on different test data. An effective solution, beyond tuning all the network hyperparameters, is using dropout, which involves discarding different parts of the data at each training epoch to prevent the network from fitting too closely to the same data. Dropout is applied both within the internal components of the LSTMs and by the dedicated layers.

The last component of the network is a fully connected (dense) layer with a linear activation in combination with a sparse categorical cross-entropy loss (set with the option “from_logits = True”), performing the final classification of the input sample. The linear activation leaves the raw scores unchanged and relies on the loss function to induce the correct behavior during training, returning as output a logits tensor. In TensorFlow, logits are the raw, unnormalized predictions output by a neural network’s final layer before any activation function is applied. These logits are essentially the scores for each class or category, indicating the model’s confidence in its predictions. Logits are essential intermediates in the classification process, providing numeric representations of the model’s confidence in each class. These raw scores enable further processing to derive meaningful predictions and are crucial for tasks like multi-class classification, where the model must distinguish between multiple categories. Following the computation of logits, then the loss function takes a vector of ground truth values and a vector of logits and returns a scalar loss for each example, encoding the logits tensor into a probability distribution. This loss is equal to the negative log probability of the true class, and thus the loss is zero if the model is sure of the correct class. Particularly, the sparse categorical cross-entropy loss function serves as the objective function to be minimized during the training process to optimize the output, and it is represented as:

(5) $$J\left(W\right)=-\frac{1}{N}\sum_{i=1}^{N}\left[y_{i}\log\left({\hat{y}}_{i}\right)+\left(1-y_{i}\right)\log\left(1-{\hat{y}}_{i}\right)\right]$$

where *W* is the set of network parameters (like node weights), *N* is the number of input samples, and $y_{i}$ and ${\hat{y}}_{i}$ are the true and predicted outputs, respectively. The Adam optimizer with a learning rate of 0.001 and the accuracy metrics are used for all the models that are tested.

## 3. Experimental Results

Several experiments were performed, with different combinations of diseases, according to their relevance from a medical point of view, as described in Section 1:

- N, AD, FTD, LBD;
  this test tries to discriminate among the main degenerative dementias (AD, FTD, LBD), collectively called DDs;
- N, AD, FTD, LBD, PSP;
  this is to try and discriminate PSP in addition to the DDs;
- N, AD, FTD, LBD, VAD;
  this is to try and discriminate VAD in addition to the DDs;
- N, DD, VAD;
  where the three DD classes are fused together;
- N, AD, FTD, LBD, PSP, VAD;
  this is the last test that comprises all the classes.

For every group of classes, several LSTM models were designed, each composed of a first LSTM layer with different output sizes to evaluate their effect on the final accuracy, and a second LSTM layer with a fixed size of 8 units.

The parameters chosen for the experiments are the following:

- Window size: 256 samples (2 s) with 50% overlap. Optimal values were established in a previous work using the same data ([17]).
- DKLT principal components retained: 50. Experimentally chosen based on relative magnitude of the most significant singular values in DKLT decomposition (Section 2.3).
- Training epochs: 100.
- Data augmentation: oversampling, with added Gaussian noise with 3% standard deviation relative to signal (Section 2.2.5). This value was experimentally chosen among a set of different values; experiments showed that a noise in the 3–10% range improved the final results.
- Data split: dataset randomly split into training and testing sets, in a 75%/25% ratio.

All the experiments were conducted using TensorFlow and Keras (v. 2.15) to train and test the models.

Table 3 summarizes the effectiveness of all prototypes. In particular, Table 3 reports the performance of the different implemented LSTM models, grouped by number of classes, in terms of model complexity and testing accuracy, specifying the number of input frames (data windows) used for the training set and the testing set.

With the increase in the number of classes to recognize, obtaining good results required increasing the size of the LSTM layers, namely from (8, 8) to (40, 8). It can be noted that there is still an increase in accuracy as the number of nodes in the input layer increases, even in cases with a small number of classes. In these latter cases, though, the accuracy improvement is less significant (e.g., for the group N, AD, FTD, LBD, the accuracy only rises from 98.6% to 98.7% for a number of nodes varying from 32 to 40).

In general, increasing the number of nodes in the first layers leads to a boost in precision but at the same time to an increase in the number of network parameters and consequently in the computational complexity. Taking into account that the number of parameters impacts the computational effort of the platform that will have to perform the classification and therefore the inference times, a good compromise seems to be the model composed of a first layer of 24 nodes, which manages, in all combinations of classes, to maintain a good accuracy (above 95%) with a reduced computational cost compared with subsequent models (about 5000 parameters).

As a matter of computational cost, it can be seen that the number of parameters is several orders of magnitude less than [22], which was examined in Section 1 as a related work, while the accuracy is still very high.

**Figure 3:**
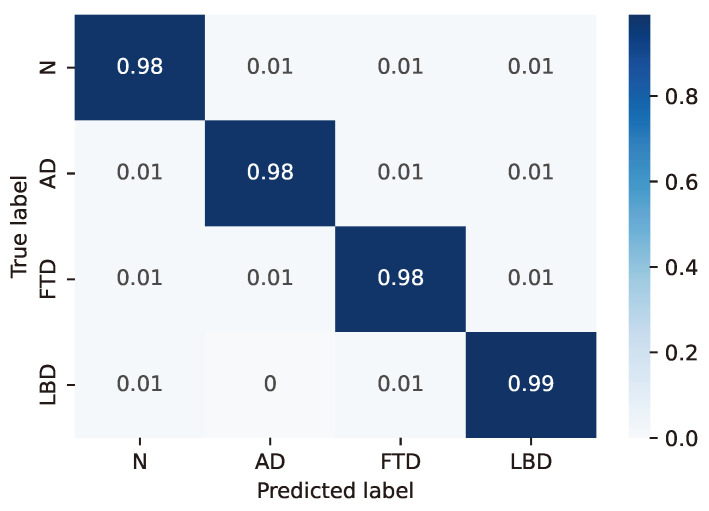
Confusion matrix for model #3 of Table 3.

**Figure 4:**
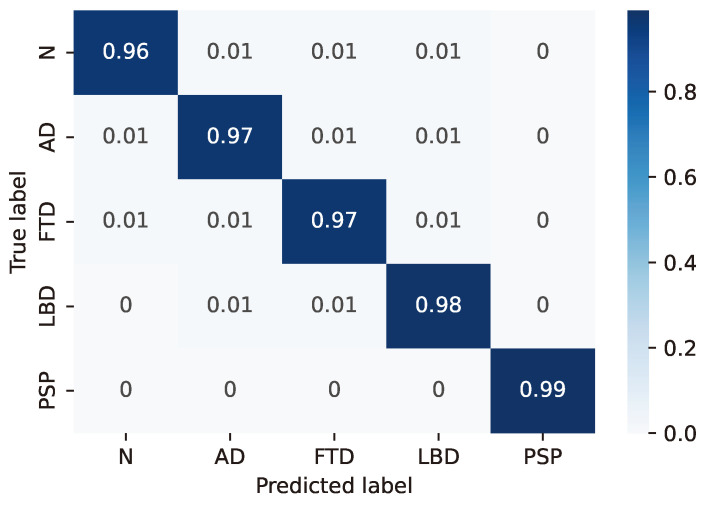
Confusion matrix for model #8 of Table 3.

**Figure 5:**
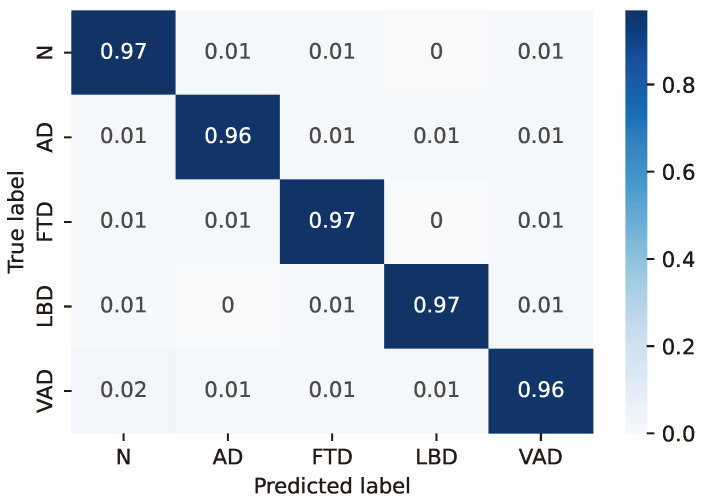
Confusion matrix for model #13 of Table 3.

**Figure 6:**
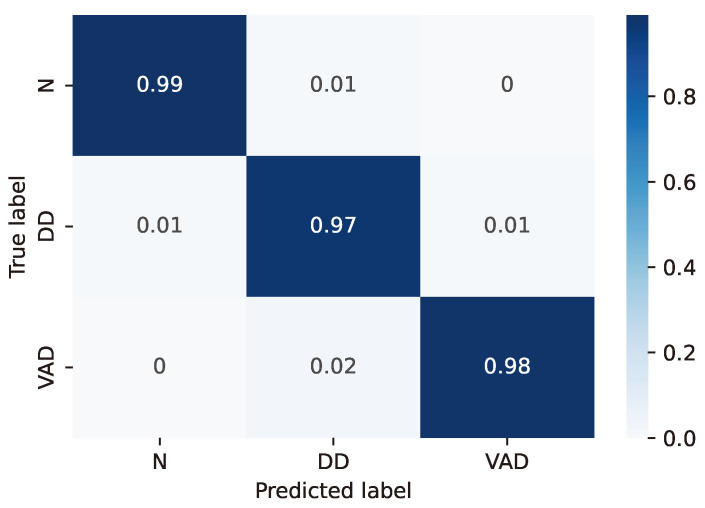
Confusion matrix for model #18 of Table 3.

**Figure 7:**
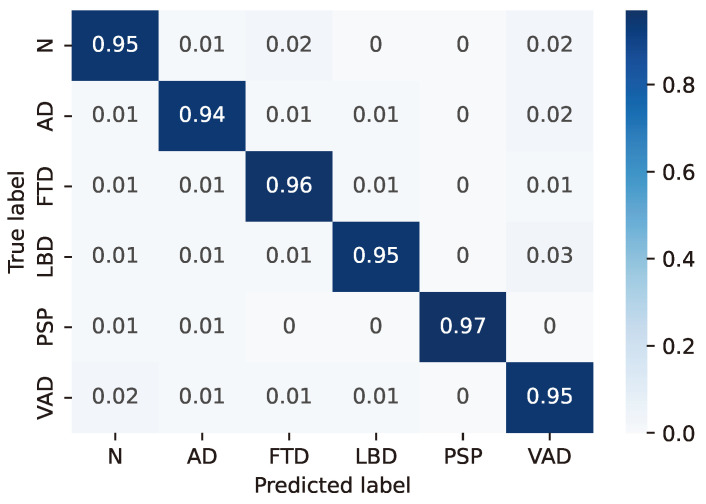
Confusion matrix for model #23 of Table 3.

For the LSTM configuration (24, 8) considered as the best case based on the previous considerations, the resulting confusion matrices are reported in detail in Figure 3, Figure 4, Figure 5, Figure 6 and Figure 7, whereas Table 4, Table 5, Table 6, Table 7 and Table 8 report the performance in terms of sensitivity, precision, and F1-score.

As a further test, for the fully comprehensive case with all the classes, a *k*-fold validation was performed: input data are randomly mixed like in the previous tests, but five tests are performed, using each time a number of the data as validation and the rest as training. Results are reported in Table 9, together with the average of the tests.

## 4. Discussion

To show the generalizability of the proposed method, its results were compared with other more traditional methods, and its performance was also evaluated on a publicly available dataset.

Regarding the comparison with more traditional statistical machine learning algorithms, the same data comprising all the six classes were tested with the *k*-nearest neighbors (kNNs) and decision tree (DT) algorithms. The input data matrices were reduced to bidimensional shapes through the same method used for the DKLT (Section 2.3).

Table 10 shows the results, which exhibit a much lower accuracy. As expected, linear statistical methods such as kNNs and DTs are not capable of solving such complex problems [46]. An accurate analysis of the effect of the classical machine learning methods applied to a subset of this dataset, both in terms of number of subjects and classes (N vs. AD), was conducted in [15], showing that only after a careful choice of the number of features, and only in few cases, the accuracy exceeded 80%, remaining around 40% in the majority of the tests conducted.

Finally, the performance of the developed model was evaluated on a public dataset used in the literature. Most of the datasets proposed in the literature contain two classes related to healthy subjects and those affected by a form of dementia (for example AD or MCI), to then perform a binary classification [23,47]. Some authors propose datasets with three classes [48,49], to then combine them two by two and then perform a binary classification.

To compare the flexibility of the proposed network on public datasets present in the literature, we used one of these three-class datasets and performed the same procedure, that is, combining the three classes into two groups. Specifically, we selected the dataset proposed by Miltiadous et al. [49,50], where two class groupings were carried out: N vs. AD and N vs. FTD. Binary classifications were performed using the Leave-One-Subject-Out (LOSO) validation method. In this approach, the feature matrices of one subject are excluded to eventually be used as the test set, while the remaining subjects constitute the training set. This process is repeated for each subject, and the final performance results are computed as the average for all subjects.

The Miltiadous dataset contains the EEG resting state-closed eyes recordings from 88 subjects in total: 36 of them were diagnosed with AD, 23 were diagnosed with FTD, and 29 were used as control subjects. Recordings were acquired using 19 scalp electrodes (Fp1, Fp2, F7, F3, Fz, F4, F8, T3, C3, Cz, C4, T4, T5, P3, Pz, P4, T6, O1, and O2) and two reference electrodes, placed according to the 10-20 international system and with a sampling rate of 500 Hz.

Table 11 shows the results obtained using the proposed LSTM network on the dataset mentioned above, following the same procedure, that is, binary classification using LOSO validation. For comparison, in [50], accuracies of 83.3% and 75.0% were achieved for the N, AD and N, FTD cases respectively, but by using a much more complicated network structure.

## 5. Conclusions

This work proposes lightweight LSTM neural networks for the classification of neurodegenerative diseases from EEG recordings. Specifically, the basic architecture is an RNN using LSTM layers as its core, which operates on the spectral representation of the data, pre-processed with filtering and data augmentation steps in order to improve the network performance.

Several experiments were performed, with different combinations of neurodegenerative diseases, according to their relevance from a medical point of view, in order to find the best models for each combination of classes. Experimental results show that a relatively simple LSTM can be used to effectively classify groups of data belonging to different classes of disease, up to six classes including the control group.

In this latter case, a simple network configured as (24, 8) LSTM units was deemed to be the best configuration, reaching an average 95.2% accuracy while only needing 5046 parameters. To the best of our knowledge, this is an excellent result considering that state-of-the-art published techniques need millions of parameters in a (1,024,512) network configuration to perform a binary (N, MCI) classification [22]. Moreover, considering all the six classes, the accuracy obtained with the proposed method overcomes that achieved by state-of-the-art machine learning methods.

The network topology and the number of parameters do not demand a high computational complexity at inference time, so that the network can be implemented on systems with limited hardware and energy resources. This, in turn, may suggest further developments investigating different kinds of hardware systems, perhaps customized to the particular working environment. Future works will focus on embedding the proposed network architecture in wearable devices for assistive diagnosis.

## Figures and Tables

**Table 1:** Dataset Consistency.

Class	Subjects	Duration (s)
Normal (N)	15	17,932
Alzheimer’s disease (AD)	20	28,586
Frontotemporal dementia (FTD)	16	21,722
Dementia with Lewy bodies (LBD)	17	22,835
Progressive supranuclear palsy (PSP)	10	10,942
Vascular dementia (VAD)	19	25,404
Total	97	127,421

**Table 2:** Details of RNN architecture (*P* = output size of LSTM 1 layer; *Q* = number of classes).

Layer	Input Size	Output Size	Parameters
LSTM 1	(–, 50, 16)	(–, 50, *P*)	$4\;(P^{2}+17\;P)$
Dropout	(–, 50, *P*)	(–, 50, *P*)	0
LSTM 2	(–, 50, *P*)	(–, 8)	4(8P+72)
Dropout	(–, 8)	(–, 8)	0
Dense	(–, 8)	(–, *Q*)	9 *Q*

**Table 3:** Experimental results for the different LSTM models.

Model #	Classes	Training Size	Testing Size	LSTM Size	N. Parameters	Testing Accuracy (%)
1				(8, 8)	1380	90.7
2				(16, 8)	2948	96.4
3	N, AD, FTD, LBD	85,698	28,566	(24, 8)	5028	98.0
4				(32, 8)	7620	98.6
5				(40, 8)	10,724	98.7
6				(8, 8)	1389	88.0
7				(16, 8)	2957	95.4
8	N, AD, FTD, LBD, PSP	107,122	35,708	(24, 8)	5037	97.6
9				(32, 8)	7629	98.6
10				(40, 8)	10,733	98.8
11				(8, 8)	1389	84.2
12				(16, 8)	2957	93.0
13	N, AD, FTD, LBD, VAD	107,122	35,708	(24, 8)	5037	96.7
14				(32, 8)	7629	97.7
15				(40, 8)	10,733	97.9
16				(8, 8)	1371	91.7
17				(16, 8)	2939	96.7
18	N, DD, VAD	164,452	54,818	(24, 8)	5019	98.0
19				(32, 8)	7611	98.9
20				(40, 8)	10,715	99.2
21				(8, 8)	1398	79.0
22				(16, 8)	2966	92.0
23	N, AD, FTD, LBD, PSP, VAD	128,547	42,849	(24, 8)	5046	95.4
24				(32, 8)	7638	97.0
25				(40, 8)	10,742	98.0

**Table 4:** Classifier performance for model #3 of Table 3.

Class	Sensitivity (%)	Precision (%)	F1-Score (%)
N	98.0	97.8	97.9
AD	97.6	98.3	98.0
FTD	97.9	98.0	97.9
LBD	98.5	98.0	98.2

**Table 5:** Classifier performance for model #8 of Table 3.

Class	Sensitivity (%)	Precision (%)	F1-Score (%)
N	96.1	98.2	97.1
AD	97.4	97.0	97.2
FTD	97.4	97.3	97.4
LBD	98.3	96.9	97.6
PSP	99.0	98.9	98.9

**Table 6:** Classifier performance for model #13 of Table 3.

Class	Sensitivity (%)	Precision (%)	F1-Score (%)
N	97.5	95.5	96.5
AD	95.8	97.1	96.5
FTD	97.4	96.9	97.1
LBD	97.3	97.5	97.4
VAD	95.5	96.4	95.9

**Table 7:** Classifier performance for model #18 of Table 3.

Class	Sensitivity (%)	Precision (%)	F1-Score (%)
N	98.9	98.3	98.6
DD	97.5	97.3	97.4
VAD	97.5	98.3	97.9

**Table 8:** Classifier performance for model #23 of Table 3.

Class	Sensitivity (%)	Precision (%)	F1-Score (%)
N	95.2	93.9	94.6
AD	94.5	95.3	94.9
FTD	95.6	94.7	95.1
LBD	94.5	97.6	96.0
PSP	97.4	98.5	98.0
VAD	95.0	92.5	93.8

**Table 9:** *k*-fold validation (k=5) for model #23 of Table 3.

Test	Accuracy (%)
1	94.3
2	95.4
3	96.2
4	95.1
5	95.1
average	95.2

**Table 10:** Results of statistical machine learning algorithms applied to the six-class full dataset.

Algorithm	Accuracy (%)
*k*-nearest neighbors	43.9
decision tree	42.0

**Table 11:** Results of binary classification from Miltiadous dataset, using LOSO validation.

Classes	Subjects	Avg. Accuracy (%)
N, AD	65	64.0
N, FTD	52	67.1

## Data Availability

The data presented in this study are available on request from the corresponding author.
